# Initiation of interdisciplinary prevention rounds: decreasing CLABSIs in critically ill children

**DOI:** 10.1017/ash.2024.55

**Published:** 2024-05-08

**Authors:** Matthew Linam, Lisette Wannemacher, Angela Hawthorne, Christina Calamaro, Patrick Spafford, Karen Walson

**Affiliations:** 1 Division of Pediatric Infectious Diseases, Emory University School of Medicine, Atlanta, GA, USA; 2 Children’s Healthcare of Atlanta, Atlanta, GA, USA; 3 Nell Hodgson Woodruff School of Nursing, Emory University, Atlanta, GA, USA

## Abstract

**Objective:**

Central line-associated bloodstream infections (CLABSIs) harm children. Insertion and maintenance bundles have significantly reduced CLABSIs, but infections still occur. The objective was to develop bedside infection prevention (IP) rounds and evaluate their impact on CLABSI rates.

**Methods:**

This quality improvement project was initiated sequentially in the neonatal intensive care unit (NICU) and pediatric intensive care unit (PICU) of a large academic children’s hospital. IP rounds, interdisciplinary discussions led by the hospital epidemiologist and unit nursing leader with the bedside nurse, occurred weekly for patients with central lines. Discussions included strategies to optimize line maintenance and identify and mitigate patient-specific infection risks. Concerns and recommendations were communicated with the clinician. CLABSIs were identified by prospective surveillance using standard definitions. The change in CLABSIs over time was analyzed using days-between-events charts (g chart).

**Results:**

IP rounds included 3,832 patients in the NICU and 1,322 patients in the PICU. Opportunities were identified to reduce line access and protect the dressing from contamination. The average days between CLABSIs in the NICU increased from 41 days to 54 days after IP rounds began. The longest time between CLABSIs was 362 days. In the PICU, the average days between CLABSIs increased from 53 to 91 days. The longest time between CLABSIs was 398 days.

**Conclusion:**

IP rounds reduced CLABSIs in the NICU and PICU by reinforcing best practices, encouraging proactive strategies, and fostering communication between members of the healthcare team.

## Introduction

Central line-associated bloodstream infections (CLABSIs) are an important source of preventable harm and excess cost in children. National Healthcare Safety Network (NHSN) data from 2006 to 2008 reported a pooled mean CLABSI rate of 3.0 per 1000 line days in pediatric intensive care units (PICUs) and 1.9–3.9 in neonates.^1^ In a large representative US inpatient database, CLABSIs in children were associated with a mean attributable cost of over $55,000 (2011 dollars) and an attributable length of stay of 19 days.^2^ In neonates, a CLABSI was associated with an attributable cost of $90,221 and an excess length of stay of 31.5 days.^2^

With the implementation of bundles focused on central line insertion and maintenance practices, hospitals have reduced CLABSIs by 40%–70%.^3–8^ Nationwide, as a result of these improvement efforts, CLABSI rates in US neonatal intensive care units (NICUs) and pediatric intensive care units (PICUs) decreased significantly between 2007 and 2012.^9^ However, data from 2013 to 2018 in both NICUs and PICUs show that CLABSI rates have plateaued.^10^ Recent data reported to the NHSN showed a significant increase in CLABSIs since the onset of the coronavirus disease 2019 pandemic.^11–13^ Staffing shortages, new staff, higher patient acuity, and disruption of routine processes resulting from the pandemic likely have amplified existing challenges contributing to the increase in CLABSIs.

In a recent study by Woods-Hill et al, the majority of surveyed nurses identified the CLABSI prevention bundle elements and understood their importance in preventing CLABSIs.^14^ However, commonly cited barriers to complete implementation included: the time required to complete the elements, competing patient care demands, and the need for additional staff assistance and patient/family refusal. Additional CLABSI prevention behaviors are primarily physician-driven such as removing unneeded lines or reducing line accesses. While nurses and physicians acknowledge each other’s roles and expertise, they continue to frequently function in silos leading to fragmented care and missed opportunities to prevent CLABSIs.^15^

In 2018, the NICU in our institution experienced an increase in CLABSIs. Based on review of CLABSIs reported in the NICU setting, we identified challenges of (1) consistently completing prevention bundle elements, (2) identifying patient-specific risk factors for infection and, (3) ineffective communication between nurses and physicians as contributing factors to the increase. To address these challenges, we developed interdisciplinary bedside infection prevention (IP) rounds designed to reinforce prevention behaviors, proactively identify and mitigate patient-specific infection risks and facilitate communication amongst the healthcare team, and evaluate the impact of IP rounds on CLABSIs.

## Methods

### Setting

This project was developed and tested in the intensive care units (ICUs) of a 370-bed tertiary academic children’s hospital that is part of a larger pediatric health system in the southeastern US. The hospital includes a 39-bed Level 4 NICU and a 56-bed PICU. Both ICUs provide care for a variety of complex critically ill medical and surgical patients. The hospital does not provide extracorporeal membrane oxygenation, cardiac care, blood and marrow transplantation, or organ transplantation. The patients requiring this level of care are transferred to or admitted to the other hospital in our system, which provides these services.

CLABSI prevention has been a system-wide safety focus since 2005. In 2005, the healthcare system implemented evidence-based prevention bundles for CLABSI (line insertion and maintenance bundles).^16^ In 2017, the hospital implemented a hygiene bundle (oral care based on patient needs, daily bath, linen change, and cleaning of high-touch surfaces). Also in 2017, the hospital updated its blood culture policy recommending both peripheral blood and central venous line cultures only in immunocompromised patients or situations in which there was concern for an infection involving the central line (ex. redness at the site or fever with use of the line). In other situations, a single peripheral blood culture was recommended.

### Infection prevention rounds

IP rounds were designed to develop strong collaborative relationships between the treating physician and clinical nurse. Bedside discussions focused on reinforcement of best practices with CLABSI bundles, provision of just-in-time teaching, working with nurses to identify patient-specific infection risks and develop mitigation strategies, and empowering the nurses to voice those concerns and strategies to the treating physician. A list of hospitalized children on the unit with a central line was generated from the electronic medical record (EMR). Information collected included the type of line, location of line insertion, date the line was placed, and the number of line accesses in the past 24 hours. Initially, all children with a central line were included in rounds. In February 2021, IP rounds focused on patients considered to be at increased risk of CLABSI. Patients were included in rounds if they had greater than the median number of line accesses (defined as PICU: 20 line accesses/24 hours, NICU: 10 line accesses/24 hours, based on internal data), had a femoral line or had the combination of a central line, Foley catheter, and endotracheal tube. Patients with a prior or current infection were not excluded from IP rounds. Additional patients were added on agreement of the IP rounding team based on any additional infection risks identified by the IP rounding team or the bedside nurse.

IP rounds were initially developed and tested in the NICU beginning in November 2018 and then implemented in the PICU in April 2019. Rounds were led by a unit nurse leader and the physician hospital epidemiologist and included the bedside nurse of each identified patient with a central line. When available, the patient’s respiratory therapist (for mechanically ventilated patients) and the unit pharmacist also participated in rounds. Rounds occurred at least once a week during weekday shifts, lasting approximately one hour in duration. To ensure consistency with rounds, a set time and day was advertised. Over time, rounds included evening and weekend shifts, but these occurred less consistently and did not include the hospital epidemiologist. The hospital epidemiologist and nurse leader met with the patient’s nurse by the patient’s room/bed space. Approximately 2–5 minutes were spent discussing each patient but varied based on the needs of the individual patient. When appropriate, caregivers were included in IP Rounds discussions. Concerns and mitigation strategies were communicated to the treating clinician by either the bedside nurse, the nurse leader, or the hospital epidemiologist.

A tool was developed by the team to standardize data collection. This tool also helped to guide the discussion and to further ensure consistent data collection by the IP rounding team. As this was an iterative process, it was revised to improve collection of additional data as needed during the implementation of the project. Initially, the tool was trialed in paper and pen format. In October 2019, the rounding tool was converted to an electronic database (Supplemental Figure), for use on a mobile platform, allowing for efficient data capture in real time. Data points documented included: central line type, line necessity, dressing integrity and concerns related to the dressing, the number of line accesses in the past 24 hours and possible strategies to reduce line access, and patient-specific CLABSI risk factors and strategies to mitigate those risks. Line accesses included all medications and infusions administered as well as associated line flushes. We captured line accesses recorded in the electronic medical record. We realize additional lab collections and medications/infusions may not have been recorded. However, this process provided consistent estimation of central line access and identified patients with frequent line access.

### Data collection and analysis

Adherence with CLABSI insertion and hygiene bundles was monitored based on documentation in the EMR. CLABSI maintenance bundle compliance was monitored based on nurse observations. Healthcare worker hand hygiene was recorded by covert direct observation by trained healthcare worker volunteers and recorded in real time using an electronic tool. Audits evaluated hand hygiene at room/bed space entry and exit and prior to contact with a central line. Data recorded during IP rounds were summarized and reported by unit beginning in October 2019 when data were collected electronically. Although discussions related to limiting line access and maintaining clean occlusive dressings occurred from the beginning of IP rounds, these were not captured in the electronic tool until February 2021. CLABSIs were identified by prospective surveillance by infection prevention using standard NHSN surveillance definitions.^17^ The monthly CLABSI rate (infections/1000 line days) was tracked for the NICU and PICU. Because of the infrequency of CLABSIs on these units and the number of months in which there were zero CLABSIs, a days-between-events chart (g chart) was used to evaluate the success of IP rounds on decreasing CLABSIs over time. The days between CLABSIs from January 2016 until the initiation of IP rounds served as baseline for each unit. Standard rules were applied to determine whether a statistically significant change had occurred.^18^

### Ethical considerations

Hospitalized children were included in IP rounds based on presence of a central line and risk of a CLABSI. Data collected on IP rounds was used for infection prevention purposes. This project was reviewed by the Children’s Healthcare of Atlanta Institutional Review Board and approved as non-human subjects research.

## Results

Since data collection began in October 2019, IP rounds included 3,832 patients in the NICU and 1,322 patients in the PICU (Table 1). Patients with prolonged hospitalizations could be rounded on multiple times depending on their risk of infection. PICC lines were the most common central venous access device in both units.

**Table 1.** Risk Factors and prevention strategies identified during infection prevention rounds for patients with central venous lines in the neonatal and pediatric intensive care units

	NICU	PICU
Type of central line^a^	*n* = 3832	*n* = 1322
Central venous line	10.0%	30.1%
PICC	88.9%	73.7%
Port	0.1%	3.0%
Umbilical vein catheter	1.6%	0.0%
Umbilical artery catheter	1.8%	0.0%
Central line still necessary	95.6% (*n* = 3829)	91.5% (*n* = 1321)
Dressing was clean, dry and intact	98.6% (*n* = 3802)	98.6% (*n* = 1321)
Presence of a peripheral IV line	28.0% (*n* = 1566)	66.9% (*n* = 695)
If yes, peripheral IV line being used for medications	43.3% (*n* = 438)	67.2% (*n* = 465)
Strategies to reduce line accesses	*n* = 1533	*n* = 676
No changes	78.7%	58.6%
Discontinue medication(s)	5.6%	10.8%
Change medication(s) to enteral route	6.6%	18.9%
Administer medication(s) via peripheral IV line	6.1%	11.1%
Change intermittent medication(s) to continuous infusion	1.3%	2.1%
Other strategy	4.1%	7.2%
Concerns related to dressing integrity or contamination	*n* = 1547	*n* = 667
No concerns	90.2%	91.5%
Difficulty maintaining integrity	2.1%	2.7%
Contamination with oral secretions	0.8%	1.0%
Contamination with stool	2.0%	1.8%
Other concern	5.6%	4.3%
Other concerns for CLABSI	*n* = 1552	*n* = 672
No additional concerns	86.5%	92.3%
Poor skin integrity	2.9%	1.2%
Mucositis	0.0%	0.3%
Other concern	11.1%	7.6%

Electronic data collection for central line type, line necessity and dressing integrity began in October 2019. All other data collection began in February 2021.

*n* is the denominator for that question.

NICU is neonatal intensive care unit.

PICU is pediatric intensive care unit.

PICC is peripherally inserted central catheter.

IV is intravenous.

CLABSI is central line-associated bloodstream infection.

a Patients may have had more than one type of central line. The tool did not capture patients with more than one of the same line type (ex 2 PICC lines).

Risk and prevention strategies were similar for the NICU and PICU. The most common strategies identified to reduce the number of line accesses included administering medications via the enteral route, discontinuing medications, or administering medications through a peripheral IV. At the time of rounds, the dressing was noted to be intact 98%–99% of the time. Patient-specific concerns related to the dressing included difficulty maintaining dressing integrity or contamination with stool or body fluids. Mitigation strategies included changing the dressing more frequently, using a different type of dressing, using a protective barrier, or moving the line to a different location. Other patient-specific risk factors included disrupted skin integrity and presence of an ostomy or surgical drain. Mitigation strategies included optimizing wound dressings and ensuring lines were kept separated from the diaper, ostomy, or drains. In general, clinicians were receptive to suggested changes, such as changing medications to the enteral route or removing or relocating a central line, but when suggestions were not implemented, the physician provided their reasoning as to why the change could not be made at that time.

Compliance with CLABSI prevention bundle data is shown in the NICU and PICU annually from 2016 through 2022 (Table 2). Hand hygiene compliance for all hand hygiene moments remained high in both units; 94%–100% in the NICU and 86%–95% range for the PICU. Adherence with the CLABSI insertion and maintenance bundles on both units remained stable in the mid to high 90% range. In the NICU, there was gradual improvement in adherence to the hygiene bundle components over time, and the overall hygiene bundle compliance increased from 70% in 2017 to 78.5% in 2022. In the PICU, for the most part, compliance with the hygiene bundle components remained stable with the exception of mouth care, which decreased from 87% in 2017 to 73% in 2022. This resulted in a decrease in overall bundle compliance from 72% in 2017 to 58% in 2022.

**Table 2.** Compliance with hand hygiene and central line-associated bloodstream infection prevention bundles in the neonatal and pediatric intensive care units from 2016 through 2022

	Compliance								
				Hygiene bundle					
Year	Hand hygiene	Maintenance bundle	Insertion bundle	Daily Bath	Mouth care	Clean high-touch surfaces	Daily linen change	Overall	Line days
**NICU**									
2016	NA	92.8% (278)	97.9% (95)	NA	NA	NA	NA	NA	NA
2017	NA	96.2% (159)	96.9% (131)	87.8%	91.7%	98.3%	84.7%	70.4%	5821
2018	100.0% (219)	100.0% (6)	98.6% (148)	89.0%	90.5%	92.6%	85.7%	67.7%	4948
2019	93.9% (932)	99.3% (304)	98.0% (152)	91.6%	91.7%	90.8%	86.7%	69.2%	4522
2020	97.4% (1857)	98.0% (354)	94.9% (156)	93.8%	93.2%	92.2%	88.1%	73.9%	5384
2021	99.5% (1326)	98.1% (258)	98.7% (156)	92.9%	95.9%	93.3%	90.7%	77.8%	5208
2022	98.8% (1467)	96.7% (180)	95.2% (165)	90.0%	97.0%	96.0%	90.4%	78.5%	5061
**PICU**									
2016	NA	93.1% (276)	97.6% (418)	NA	NA	NA	NA	NA	NA
2017	NA	93.2% (206)	98.9% (348)	93.4%	86.6%	97.3%	86.7%	71.9%	3903
2018	85.9% (333)	95.6% (205)	98.7% (397)	95.2%	81.6%	88.9%	86.9%	64.4%	3336
2019	88.6% (1224)	99.2% (130)	98.8% (425)	95.2%	77.1%	88.1%	88.2%	63.0%	4052
2020	94.8% (842)	97.6% (127)	98.6% (348)	94.6%	72.2%	89.3%	88.0%	60.4%	3231
2021	92.9% (860)	98.0% (100)	97.4% (385)	94.6%	73.5%	90.9%	87.2%	59.5%	3940
2022	91.4% (592)	91.7% (72)	94.4% (356)	91.7%	73.1%	92.2%	85.3%	57.9%	3728

CLABSI is central line-associated bloodstream infection. NA is not applicable. CHG is chlorhexidine gluconate. NICU is neonatal intensive care unit. PICU is pediatric intensive care unit.

For hand hygiene, maintenance bundle and insertion bundle, data are shown as the annual percent compliance (total number of observations). For the hygiene bundle components, data are shown as percent compliance based on the annual line days.

Healthcare worker hand hygiene was monitored by covert observations made by healthcare worker volunteers during routine care and recorded electronically beginning in 2018.

The CLABSI insertion and maintenance bundles were implemented in 2005.

The maintenance bundle included: hand hygiene and donning gloves prior to accessing the line, scrubbing the line access point prior to access, assessing and documenting that the dressing is clean and intact as well as date of last dressing change, assessing line necessity, daily CHG bath and connecting tubing to the line aseptically.

The hygiene bundle was implemented in 2017 and included: daily bath, appropriate mouth care each shift based on the patient’s age, cleaning high-touch surfaces each shift, and daily linen change.

Compliance with the insertion bundle and hygiene bundle was monitored based on documentation in the electronic medical record. Compliance with the maintenance bundle was based on nurse observations.

**Figure 1.**
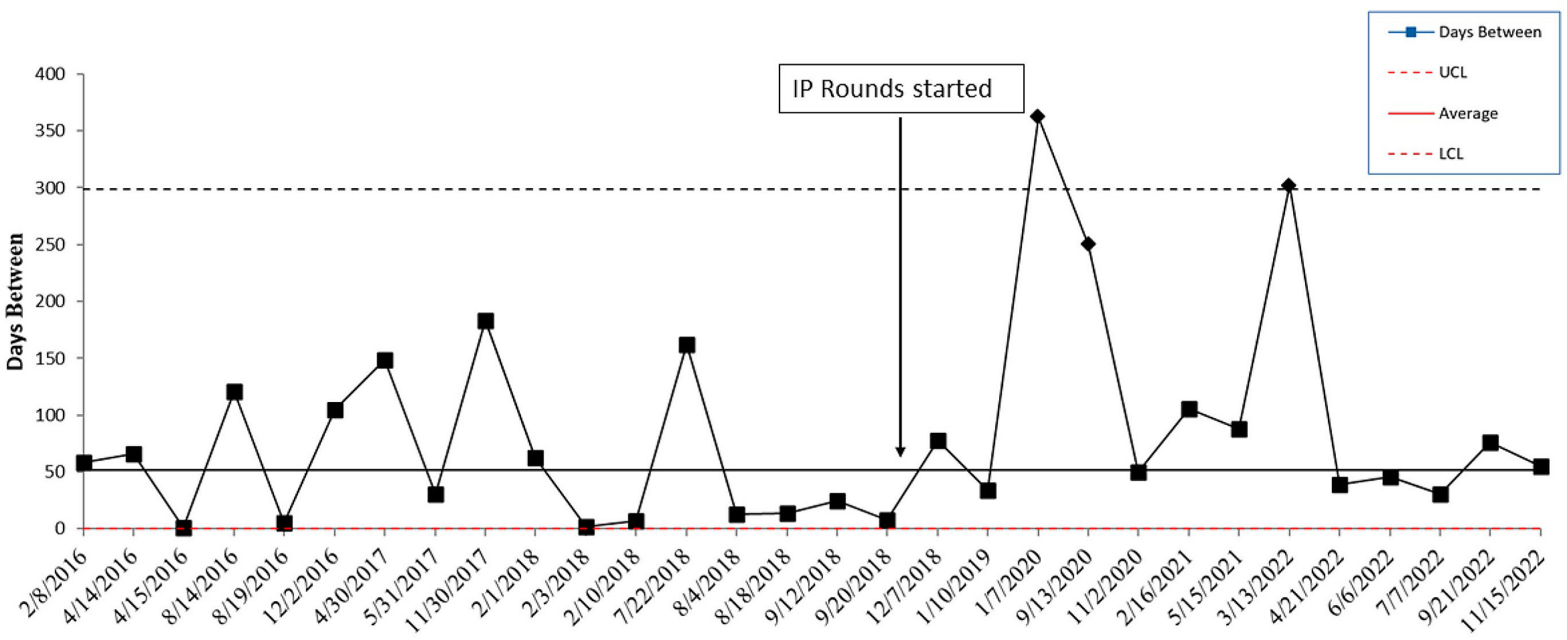
Statistical process control chart showing the change in the days between central line-associated bloodstream infections in the NICU before and after implementation of infection prevention rounds. NICU is neonatal intensive care unit. IP rounds are infection prevention rounds. CLABSI is central line-associated bloodstream infection. The Y axis is days between CLABSIs. The X axis denotes the dates of CLABSIs.

**Figure 2.**
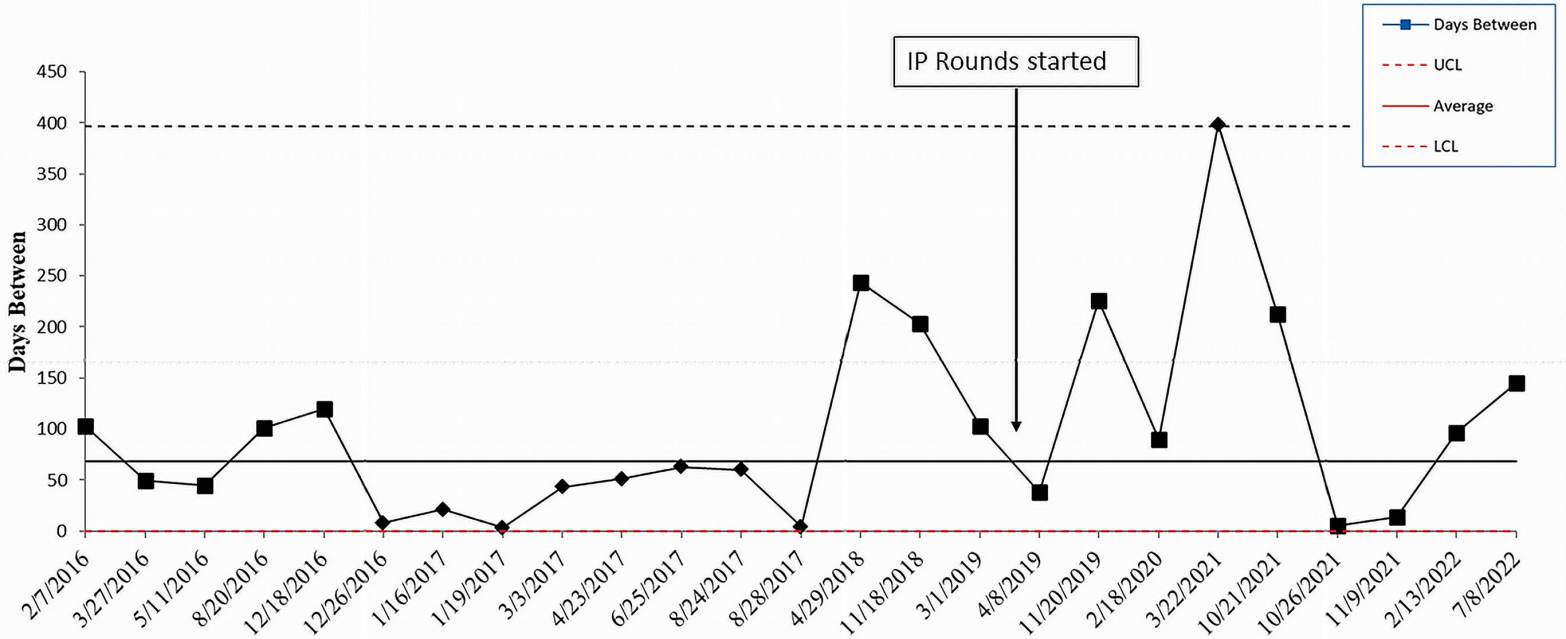
Statistical process control chart showing the change in the days between central line-associated bloodstream infections in the PICU before and after implementation of infection prevention rounds. PICU is pediatric intensive care unit. IP rounds is infection prevention rounds. CLABSI is central line-associated bloodstream infection. The Y axis is days between CLABSIs. The X axis denotes the dates of CLABSIs.

The baseline monthly CLABSI rate/1000 line days in the NICU (January 2016 through October 2018) was 0.99. After IP rounds began in the NICU in November 2018, the CLABSI rate decreased to 0.51 (48.5% decrease). The baseline monthly CLABSI rate in the PICU (January 2016 through March 2019) was 1.13. This decreased to 0.67 (41% decrease) after IP rounds began in April 2019. The average days between CLABSIs in the NICU increased from 41 days to 54 days (Figure 1). The longest time between CLABSIs was 362 days. In the PICU, the average days between CLABSIs increased from 53 to 91 days (Figure 2). The longest time between CLABSIs was 398 days.

## Discussion

This four-year IP rounds project successfully demonstrated how a team approach to consistently discuss key components of CLABSI prevention can improve infection rates in critical care areas such as the NICU and PICU. During this time, there was a sustained increase in the days between CLABSIs in both units. Conversations during rounds identified opportunities to optimize prevention bundles and mitigate patient-specific infection risks, and improved communication between the healthcare team.

A similar project in a single PICU implemented physician and nurse co-led weekly rounds focused on CLABSI maintenance bundle compliance. Rounds identified 25% of lines with high daily line access numbers and 10% of lines in place for an excessive duration highlighting opportunities for improvement.^19^ They were able to improve both physician and nurse line maintenance practices and reduce the CLABSI rate by almost 19% over four months.^19^ IP rounds shared some similarities with rounds developed in this project, but IP rounds not only discussed components of the maintenance bundle but also encouraged nurses to identify other potential infection risks and develop solutions. Our project reduced the CLABSIs in both the NICU and PICU and sustained this improvement for over four years. These outcomes are notable as they occurred at a time when CLABSI rates were increasing in children across the country.^11,13^

Patient Safety Leadership WalkRounds^TM^ is a healthcare management tool to facilitate leaders promoting safety, listening to frontline worker concerns, supporting accountability, and resourcing risk mitigation.^20^ There are three necessary components of effective WalkRounds: rounds need to occur on a regular basis, identified safety issues are addressed and feedback on these actions is provided to frontline staff. WalkRounds with feedback is considered a marker of effective WalkRounds. In a study of over 16,000 healthcare workers, settings where WalkRounds with feedback occurred more frequently reported higher scores related to safety culture and workforce engagement and lower burnout.^21^ While IP rounds were not directly fashioned after WalkRounds, a number of similarities exist in their design and implementation. IP rounds created a similar framework for weekly collaborative discussions between physicians and nurse leaders and bedside nurses focused on preventing CLABSIs.

Improved teamwork has been associated with improved patient outcomes including reduced CLABSIs.^22^ In a study of 44 NICUs, physician/nurse teamwork was independently associated with HAIs such that for every 10% increase in respondents reporting good teamwork, there were 18% lower odds of very low birth-weight infants contracting a healthcare-associated infection.^23^ Teamwork survey items most associated with HAIs were physician-nurse coordination, communication, and perceptions of nurse input. Although the impact on teamwork was not measured, IP rounds incorporated these teamwork components into the bedside conversations and achieved a sustained reduction in CLABSIs.

Prior to IP rounds, the NICU and the PICU demonstrated high levels of hand hygiene compliance and adherence to the insertion and maintenance bundles. Despite this, CLABSIs continued to occur. This suggests lapses in prevention bundle compliance not captured by the data, other risk factors not addressed by the bundles, or both. Both units demonstrated a reduction in CLABSIs after the initiation of IP rounds; although, the primary reason for this was likely different for each unit. In the NICU, there were fewer identified opportunities to reduce patient-specific risk factors such as reducing line access. However, adherence to the hygiene bundle, especially mouth care, consistently improved after rounds began. In the PICU, recorded hygiene bundle compliance decreased over time, mainly due to decreased mouth care. Decreased mouth care was likely a combination of failure to document completed mouth care and failure to perform mouth care. New staff and high patient acuity probably contributed to both situations. In the PICU, there were more opportunities identified to mitigate patient-specific risks for infection. Between the two units, opportunities to decrease the number of times the central line was accessed were identified in 40% of patients. Additionally, IP round discussions led to many of the lines identified as no longer necessary being removed. Our findings suggest that the benefit of IP rounds on CLABSI reduction likely varies based on the needs of the unit.

There were a couple of limitations with this project that should be noted. This project was implemented in the NICU and PICU of a single children’s hospital and may not have equal success based on regional and cultural differences of other healthcare institutions. Although no other CLABSI interventions occurred during interdisciplinary rounds, factors such as additional nursing education or laboratory training may have affected outcomes.

Implementation of IP rounds in the NICU and PICU resulted in a sustained decrease in CLABSIs. IP rounds created a replicable sustainable framework for purposeful discussions with bedside nurses to reinforce best practices and identify infection risks in their patients and implement strategies to mitigate those risks. Engagement of both nurses and physicians was key for successful implementation. Future projects include understanding the impact of IP rounds in other ICUs and expanding these types of purposeful conversations to try to reduce other healthcare-associated infection and safety issues.

## Supporting information

Linam et al. supplementary material Linam et al. supplementary material

## Supplementary material

For supplementary material accompanying this paper visit http://doi.org/10.1017/ash.2024.55. click here to view supplementary material
