# Supplementary figures and images for: Initiation of interdisciplinary prevention rounds: decreasing CLABSIs in critically ill children
Source: Antimicrob Steward Healthc Epidemiol. 2024 May 8;4(1):e80. doi: 10.1017/ash.2024.55 (PMC11077607; doi:10.1017/ash.2024.55)

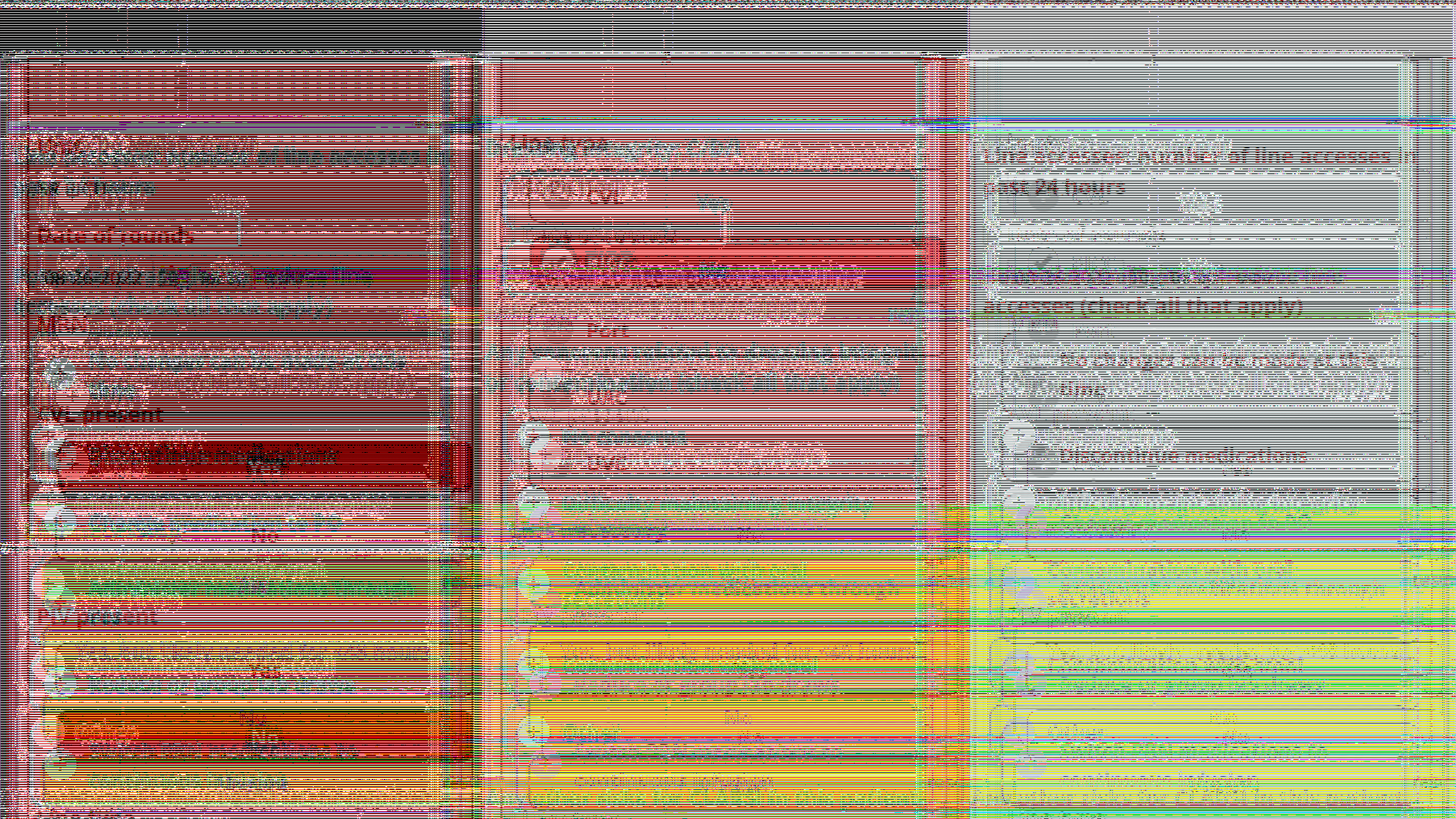

Supplement: Linam et al. supplementary material [file S2732494X2400055Xsup001.tif]
